# Trophic Factor-Induced Activity ‘Signature’ Regulates the Functional Expression of Postsynaptic Excitatory Acetylcholine Receptors Required for Synaptogenesis

**DOI:** 10.1038/srep09523

**Published:** 2015-04-01

**Authors:** Collin C. Luk, Arthur J. Lee, Pierre Wijdenes, Wali Zaidi, Andrew Leung, Noelle Y. Wong, Joseph Andrews, Naweed I. Syed

**Affiliations:** 1 Hotchkiss Brain Institute, Faculty of Medicine, University of Calgary, Alberta, Canada

## Abstract

Highly coordinated and coincidental patterns of activity-dependent mechanisms (“fire together wire together”) are thought to serve as inductive signals during synaptogenesis, enabling neuronal pairing between specific sub-sets of excitatory partners. However, neither the nature of activity triggers, nor the “activity signature” of long-term neuronal firing in developing/regenerating neurons have yet been fully defined. Using a highly tractable model system comprising of identified cholinergic neurons from *Lymnaea*, we have discovered that intrinsic trophic factors present in the *Lymnaea* brain-conditioned medium (CM) act as a natural trigger for activity patterns in post- but not the presynaptic neuron. Using microelectrode array recordings, we demonstrate that trophic factors trigger stereotypical activity patterns that include changes in frequency, activity and variance. These parameters were reliable indicators of whether a neuron expressed functional excitatory or inhibitory nAChRs and synapse formation. Surprisingly, we found that the post- but not the presynaptic cell exhibits these changes in activity patterns, and that the functional expression of excitatory nAChRs required neuronal somata, *de novo* protein synthesis and voltage gated calcium channels. In summary, our data provides novel insights into trophic factor mediated actions on neuronal activity and its specific regulation of nAChR expression.

Activity-dependent mechanisms are implicated in synaptic connectivity of both excitatory and inhibitory synapses^1^,^2^,^3^ as well as synaptic plasticity^4^,^5^. However, unequivocal evidence for the direct involvement of electrical activity in coordinating precise patterns of neuronal connectivity is still lacking. For instance, while both spontaneous and experience-driven neuronal activity patterns are considered necessary for the refinement of synaptic connectivity in the visual system^6^,^7^,^8^,^9^,^10^, as well as transmitter release and receptor expression^11^,^12^,^13^,^14^,^15^, the precise role of electrical activity and the underlying mechanisms remain largely unknown. This lack of fundamental knowledge in the field of neurodevelopment owes its existence to the fact that direct, controlled, non-invasive and simultaneous measurements of electrical activity patterns of both pre- and postsynaptic neurons cannot be achieved, nor can they be manipulated experimentally over an extended period through conventional electrophysiological techniques. Further, because direct, long-term intracellular recordings from both pre- and postsynaptic neurons are not feasible in most model systems, only extracellular field recordings or short-term ion channel monitoring have been used to deduce the involvement of electrical activity during neurodevelopment^16^,^17^,^18^.

Trophic factors and their receptors have recently been shown to affect neuronal excitability by modulating ion channel function, transmitter release or synaptic plasticity, contributing to synapse assembly and efficacy^19^. Mechanistically, it is believed that trophic factor-induced synapse formation and synaptic plasticity may invoke activity-dependent processes^20^,^21^, though the precise target sites for trophic factor actions and the underlying steps remain poorly defined. Previous studies in *Lymnaea* have demonstrated that extrinsic trophic factors derived from brain conditioned medium (a trophic factor laden culture media) are necessary for excitatory but not inhibitory synapse formation^22^,^23^,^24^,^25^ and that this synaptogenesis likely involves trophic factor mediated changes in neuronal excitability.

Here, we utilized neuron-chip interfacing technology to obtain non-invasive recordings from individual neurons with high temporal resolution. We demonstrate that following CM addition the activity pattern of postsynaptic neuron left pedal dorsal one (LPeD1) undergoes a shift from inconsistent, high variance activity to a more consistent, low frequency variance over a period of 10 hours. The neurons that exhibited this activity pattern were more likely to express functional excitatory nAChRs than those that did not. While we are cognizant that in this study we did not perform quantitative analysis of receptor expression levels, the term receptor “expression” is used here in the context of the phenotype response of cells (excitatory, inhibitory or biphasic) as determined electrophysiologically. Hence, in this study, we report on a novel mechanism whereby a specific activity “signature” is deemed necessary to prime the postsynaptic neuron for excitatory synapse formation and synaptic plasticity, through the functional expression of excitatory nAChRs.

## Results

### Trophic factors trigger activity in the post- but not the presynaptic cell

Trophic factors present in *Lymnaea* CM are required for excitatory but not inhibitory synapse formation^24^. The CM-induced effects are also mimicked by *Lymnaea* epidermal growth factor (LEGF) purified from *Lymnaea* albumin glands, human EGF and TGF alpha. More recently, the *Lymnaea* EGF receptor was cloned and its knock down prevented both CM and EGF-induced excitatory synapse formation^26^. Here, to ensure that a full complement of all naturally occurring trophic molecules secreted intrinsically by the brain tissue are present, we opted to use *Lymna*ea CM. It is important to note that all CM-induced effects reported here are also mimicked by LEGF, albeit less consistently.

**Figure 1:**
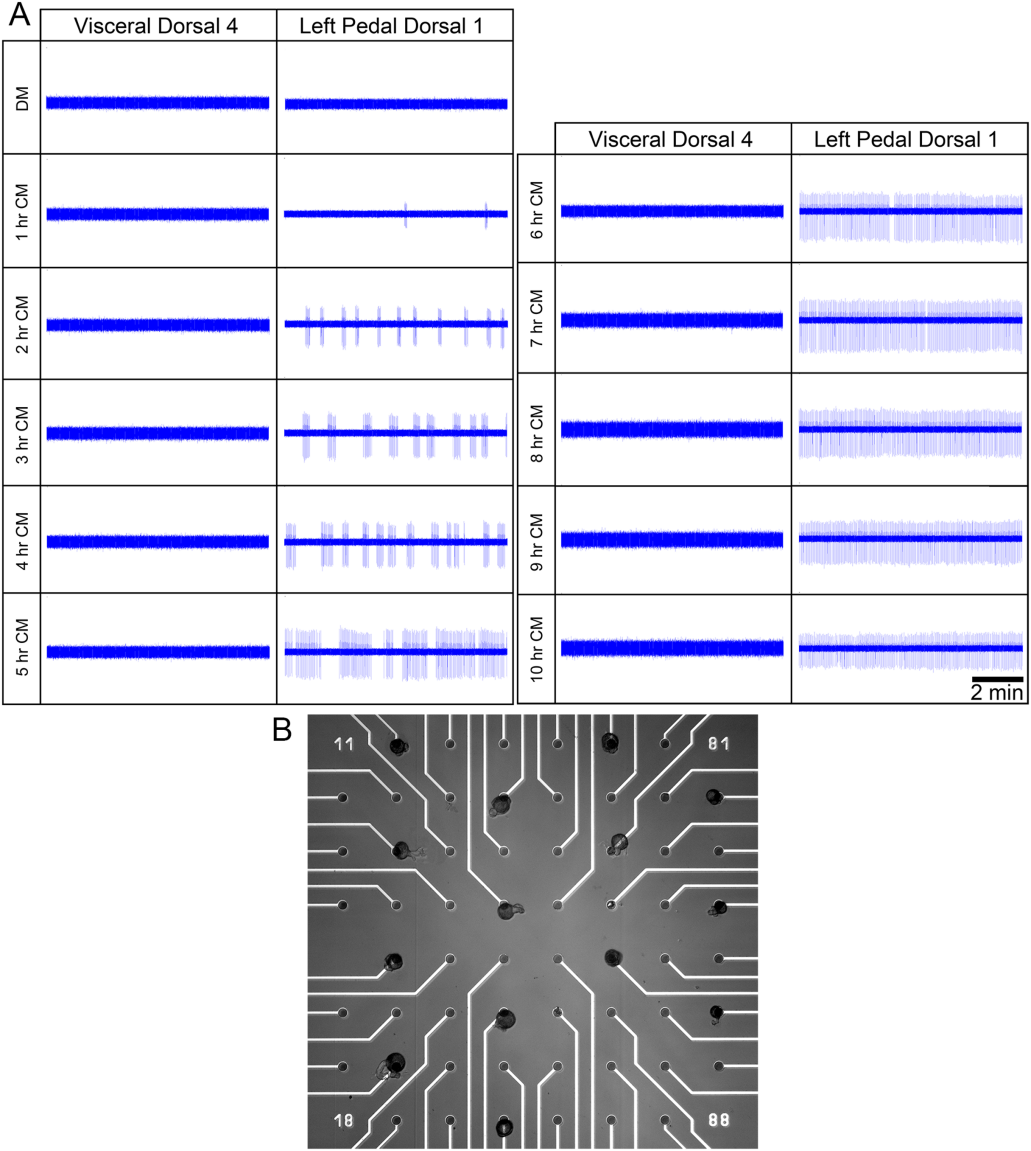
Activity of VD4 and LPeD1 neurons following exposure to CM. Neuronal electrical activity was observed over a 12 hours period using microelectrode arrays. VD4 and LPeD1 neurons were cultured on microelectrode arrays in DM (without trophic factors) overnight and CM (with trophic factors) was added the next day following 2 hours of control DM recordings. (A) A sample 5 minutes trace obtained during DM control and every hour following CM introduction is shown. (B) A representative figure of neuronal placement on the chip. The first three columns on the left are LPeD1 neurons, and the last column on the right shows VD4 neurons.

To test for the effects of CM on isolated presynaptic VD4 and postsynaptic LPeD1 neurons, cells were placed on individual microelectrode array (MEA) electrodes overnight in DM (Fig. 1B). Twelve to eighteen hours post-isolation, all cells were simultaneously recorded for two hours. This initial two-hour period served as control recordings for VD4 and LPeD1 neurons prior to CM exposure. In all cases, neither LPeD1 nor VD4 exhibited any persistent activity during these control recording periods (Fig. 1A). Next, CM was exchanged with DM and an additional 10 hours of recordings were conducted. Whereas CM addition did not have any significant effect on VD4, the activity in LPeD1 increased significantly (Fig. 1A). Specifically, within the first hour, small bursts of action potentials (~2–3 AP/burst) were recorded in LPeD1. In the second hour, the size of the bursts (~4–5 AP/burst) as well as the frequency of the bursting events increased. The third and fourth hour revealed a further increase in the duration of bursting activity (~7–10 AP/burst) with the fifth hour showing larger bursts of activity that were comprised of 15–30 action potentials per event. From the sixth to the tenth hour, LPeD1 neurons exhibited an almost persistent level of action potential firing, marked by brief periods of inactivity. Conversely, no change in activity was observed was observed in VD4 over the 10 hour period. Hence, trophic factors triggered activity in postsynaptic LPeD1 neurons, but not presynaptic VD4. As a control experiment, DM was exchanged with fresh DM to rule out mechanical artifacts that may have caused neuronal excitability. We saw no change in activity (data not shown). We next performed quantitative measurements of LPeD1 activity.

### Trophic factors selectively trigger a progressive increase in LPeD1 activity over a 10 hour period

**Figure 2:**
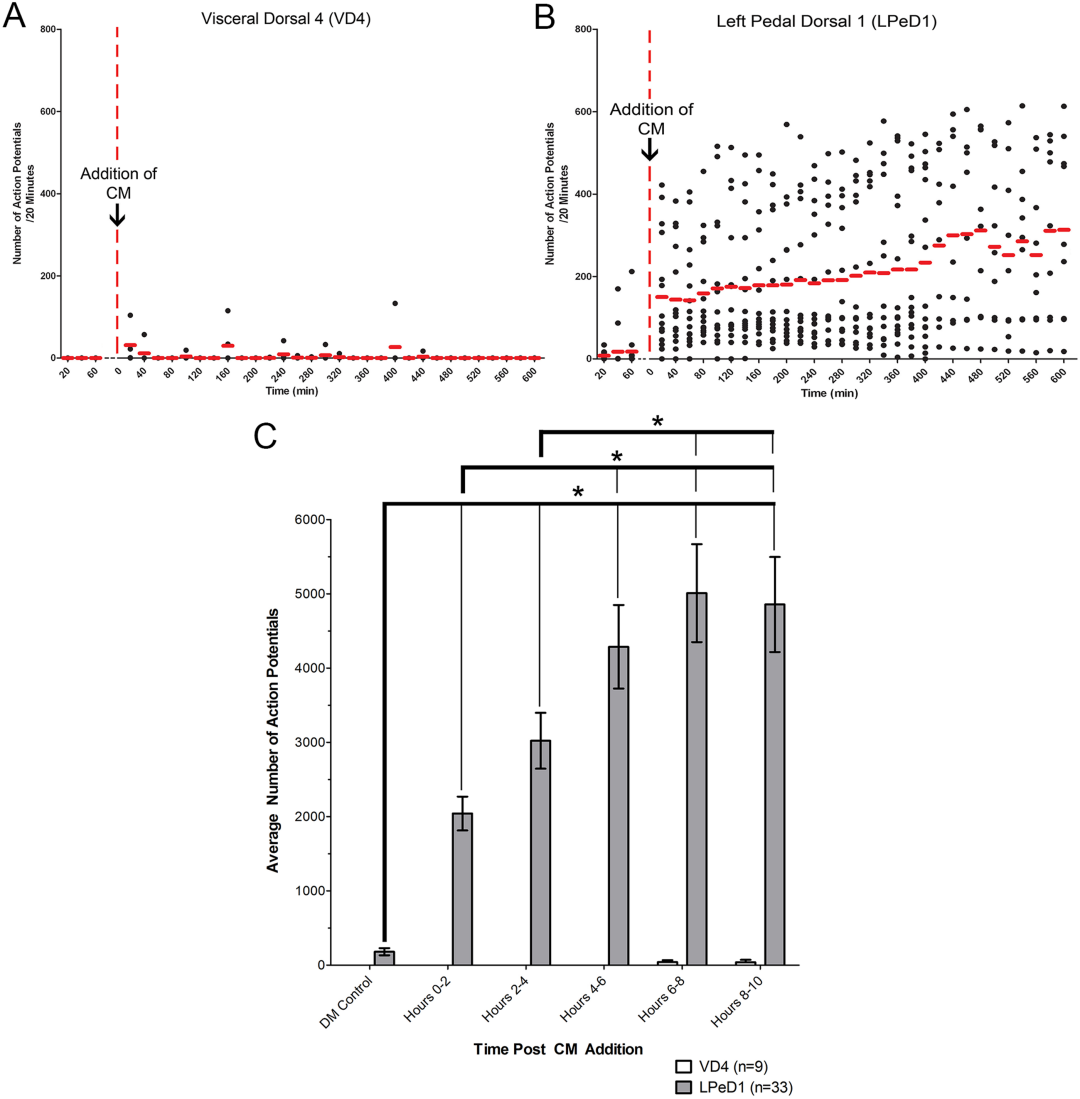
LPeD1 and VD4 activity in 20 minute and 2 hour bins following exposure to CM. Neuronal activity is shown as observed over a 10 hour period using microelectrode arrays. VD4 and LPeD1 neurons were cultured on the microelectrode arrays in DM (without trophic factors) overnight, and CM (with trophic factors) was added the next day following 2 hours of control DM recordings. Activity from (A) VD4 and (B) LPeD1 isolated neurons is shown in the scatterplot with the red line designating the mean number of action potentials for 20 minute bins. The activity was then (C) further binned into two hour periods for both VD4 and LPeD1 neurons and a one-way ANOVA showed significant differences at different time periods, with a maximal activity plateau reached between 5–10 hours. Note a rapid rise in activity in LPeD1 following CM addition as compared to that observed in VD4, which did not exhibit a similar response. Significant differences are denoted with an asterisk (*) and significance was assumed if p < 0.05.

To quantify CM-induced activity increases, we analyzed the total number of action potentials in LPeD1 neurons in 20 minutes bins, using VD4 neurons as a control. Prior to the addition of CM, VD4 and LPeD1 neurons were relatively quiescent (Fig. 2A, B). Upon CM addition, control VD4 neurons did not show any change in activity, whereas LPeD1 neurons exhibited a rapid increase in spiking, which ramped up over the ten hour recording period (Fig. 2A, B). To determine statistical trends in activity changes, the data were binned into two-hour blocks. We found that following CM addition, the immediate activity increase in the first 0–2 hours was significantly greater compared to control. This trend continued until hours 6–8, at which point activity peaked and maintained a steady level until the termination of recording at ten hours (Fig. 2C; n = 33). All levels of activity following CM addition were significantly greater than when cells were maintained in DM (p < 0.05). Activity during the 4–6, 6–8 and 8–10 hours increased significantly from the initial 0–2 hours (p < 0.05). Furthermore, activity at hours 6–8 and 8–10 was also significantly greater than hours 2–4 (p < 0.05). These data thus suggest that CM induced activity in LPeD1 increases until a plateau is reached during hours 6–8 and 8–10 post addition of the CM. The peak level of activity occurred at hours 6–8 following the CM addition (5011 ± 660) and did not change significantly at hours 8–10 (p > 0.05). In all instances observed, a large increase in LPeD1 activity was observed immediately following CM addition (n = 33), while VD4 remained unresponsive (Fig. 2C; n = 9).

### nAChR phenotype in LPeD1 is correlated with different trophic factor induced activity patterns

**Figure 3:**
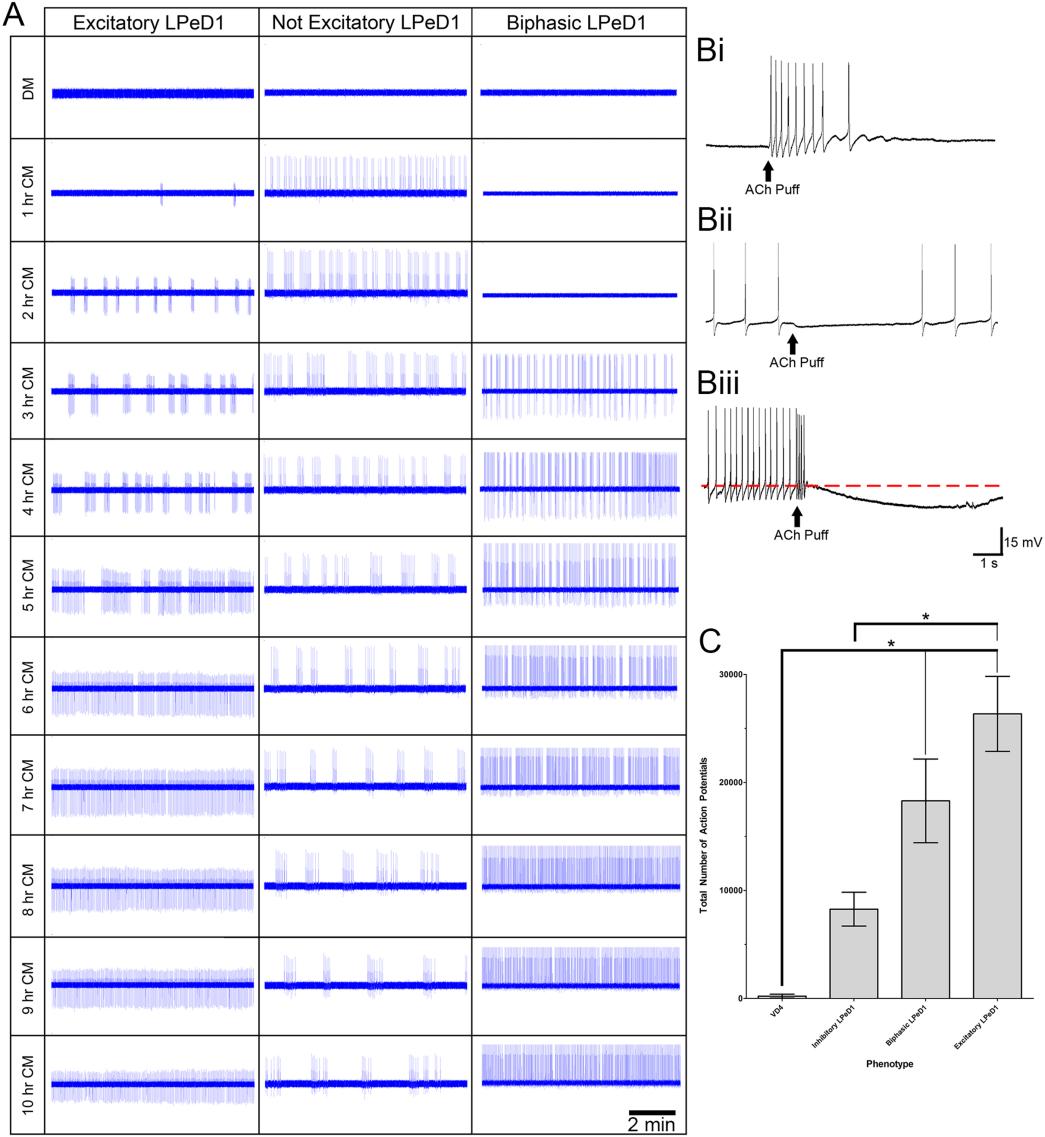
Total number of action potentials versus final functional AChR phenotype. (A) The activity triggered in response to conditioned media was different for all three phenotypes of isolated LPeD1 neurons. The figure shows 10 minutes samples of activity observed at various time points following CM exposure. Three different LPeD1 phenotypes were deciphered by neuronal responses to exogenously applied acetylcholine, which elicited 3 different effects, excitatory (Bi), inhibitory (Bii), and biphasic (Biii; elements of both excitation and inhibition). In (Bi), application of acetylcholine excited the cell, eliciting action potentials and increasing the frequency of activity. In (Bii), acetylcholine inhibited the cell, stopping the firing of action potentials and induced a decrease in neuronal membrane potential. In (Biii), acetylcholine elicited an action potential that was followed by a subsequent decrease in membrane potential (a pre-application potential line is shown as a red dotted line to aid in visualization). Acetylcholine applications are indicated by a solid arrow. The total LPeD1 activity is stratified according to neuronal response to acetylcholine application (C). It can be seen that cells with excitatory response had higher levels of activity than those that exhibited an inhibitory response. Significance is denoted with an asterisk (*) and is assumed if p < 0.05.

To test for the presence of functional excitatory nAChRs, intracellular recording were coupled with exogenous acetylcholine (ACh) applications. Similar to our previous studies^25^, LPeD1 neurons cultured in DM were inhibitory. However, following CM exposure, a majority of the LPeD1 cells switched from inhibitory to excitatory (15/33) (Fig. 3Bi). In particular, ACh application resulted in an excitatory response, which triggered action potentials in LPeD1. However, in a smaller population of cells, either no or an incomplete changeover was observed, with some LPeD1 neurons remaining either inhibitory (9/33) (Fig. 3Bii) or changing into an intermediary phase known as the “biphasic” response (9/33; elements of excitation and inhibition) (Fig. 3Biii). All three different end-point phenotypes were stratified and correlated with their activity pattern (Fig. 3A). In cells exhibiting an inhibitory response to ACh, an immediate increase in activity was noted following the CM exposure, which remained unchanged over time. In contrast, in excitatory LPeD1 neurons, there was a pattern of increasing activity over time, which eventually transitioned into more consistent firing that persisted throughout the experiment. Conversely, in biphasic neurons, a similar trend towards increased activity seen in excitatory LPeD1 neurons was observed. This response to CM, however, appeared to be delayed in some neurons whereby the activity onset occurred approximately three hours following the addition of CM. Additionally, the total activity over a 10 hour time window was also less than that seen in neurons exhibiting an excitatory response. This suggests that neurons exhibiting a biphasic phenotype showed characteristics intermediate of an inhibitory and excitatory response, which could potentially switch over to complete excitation given the additional time.

To further quantify this difference in activity patterns in the above three phenotypes, we examined the total number of action potentials over a 10 hour period following the trophic factor addition and correlated them to the phenotype observed at the end of 10 hours (Fig. 3C). We found that LPeD1 neurons that expressed functional excitatory nAChRs were significantly more active than those cells that expressed functional inhibitory nAChRs (both inhibitory LPeD1, VD4). However, there was no significant difference in activity between inhibitory LPeD1 and control VD4 (p > 0.05) or between biphasic LPeD1 and excitatory LPeD1 (p > 0.05). We did, however, observe a trend towards increased activity from inhibitory to biphasic to excitatory cells. These data suggest that the level of activity, specifically the total number of action potentials, is strongly correlated with an ultimate phenotype that an LPeD1 neuron exhibits following CM exposure.

**Figure 4:**
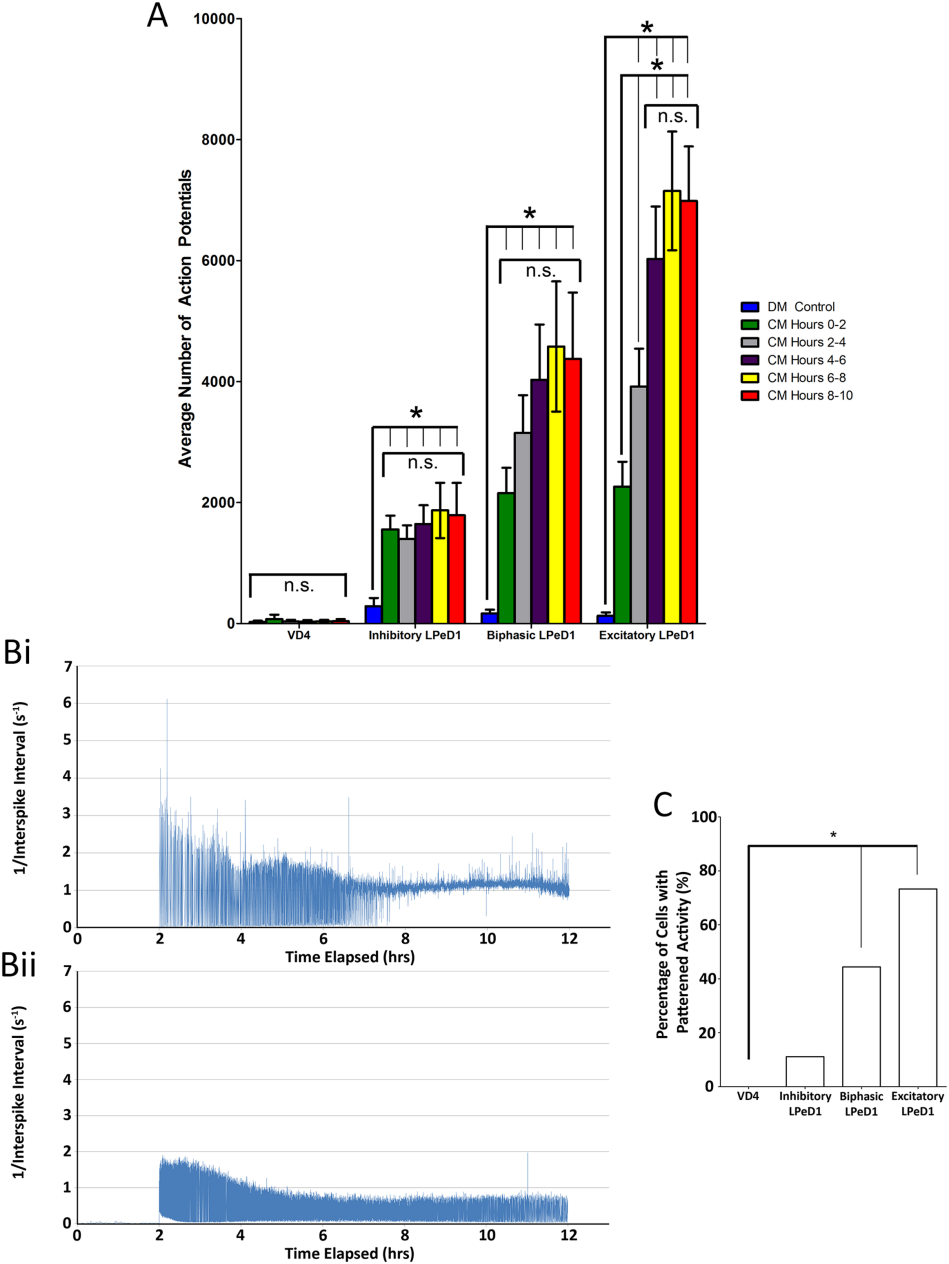
Neuronal activity in inhibitory, biphasic and excitatory LPeD1 cells and the corresponding activity pattern expressed as 1/interspike interval. (A) Neuronal electrical activity observed over a 12 hour period of VD4 and LPeD1 cells that responded to acetylcholine with inhibitory, biphasic, and excitatory responses. VD4 and LPeD1 neurons were cultured overnight on MEAs in DM and control recordings were obtained in DM followed by CM exposure. The responses of LPeD1 cells to ACh were tested 10 hours following CM addition. Each measure of activity represents the average number of action potentials recorded per a 2 hour period. Note the different responses of LPeD1 cells exposed to CM among the various different LPeD1 phenotypes. Next, to examine activity over an extended period of time, the interspike interval (ISI; time elapsed between two action potentials) was calculated and the inverse of the ISI was taken to give a measure of frequency. Using this protocol, we examined the firing pattern and found that a characteristic of (Bi) excitatory LPeD1 neurons was a progression of activity towards a tightly regulated firing rate of approximately 1 Hz, while the firing frequency of (Bii) inhibitory cells did not undergo this change in pattern and neurons continued to fire at a highly variable rate. After fingerprinting this pattern, we correlated the patterned activity with receptor expression using blinded scoring. It can be seen that (C) excitatory receptor expressions is significantly correlated with patterned activity. Significant differences are denoted with an asterisk (*) and significance is assumed if p < 0.05. Non-significantly different groups were designated with “n.s.”.

Next, to further characterize differences in activity between the three phenotypes, we examined activity trends over the 10 hour period. A unique trend for each phenotype was observed (Fig. 4A). For instance, in LPeD1 neurons expressing only functional excitatory receptors, an immediate increase in activity following CM addition was noted in hours 0–2 after the CM addition, which continued to increase significantly before peaking and plateauing at hours 4–6. In LPeD1 neurons expressing a biphasic phenotype, an initial trend, similar to that of excitatory LPeD1 was noted (hours 0–4). However, the peak levels of activity were less than those observed in excitatory LPeD1 neurons. In contrast, in LPeD1 cells exhibiting an inhibitory response, there was a similar immediate increase in activity after the CM addition at hours 0–2. However, the subsequent activity remained flat with no further increases following hours 0–2. These data thus suggest that the nAChR receptor phenotype in LPeD1 appears to be correlated with progressive increases in activity following the trophic factor addition. LPeD1 cells with excitatory nAChRs not only fired more action potentials but also exhibited a specific trend as compared to their counterparts expressing inhibitory nAChRs. These data also suggest that a specific pattern of activity is likely required for trophic factor induced functional excitatory receptor expression.

### Patterned activity was correlated with excitatory nAChR expression

To provide better characterization of the activity patterns over time, we captured individual action potential and examined a “time stamp” for its occurrence. With these time logs, we calculated the interspike interval (ISI; time between two adjacent action potentials). We then measured the inverse of each ISI to provide a measure of frequency that corresponded directly with two adjacent action potentials. These ISIs were visualized on a scatterplot allowing us to determine variability, frequency and change in activity (Fig. 4Bi, Bii).

Using the above approach, cells were deemed to have a “patterned” activity after 10 hours of trophic factor exposure if, *(1)* there was a progression from high to low variability in frequency, and *(2)* the final frequency of activity ranged from ~1–1.5 Hz (Fig. 4Bi). In cells that were deemed to be “non-patterned”, the action potential frequency continued to show *(1)* variability in their inverse ISI values as compared to the neurons with patterned activity (Fig. 4Bii; p < 0.05) and *(2)* did not achieve a consistent ~1–1.5 Hz activity during the latter half of the recording.

With these criteria, we examined whether the “patterned” activity could be generalized to all LPeD1 neurons. We performed blind scoring of each cell's activity using the inverse ISI analysis and predicted the final cell phenotype (excitatory, inhibitory) using the above two criteria (Fig. 4C). We found that the expression of “patterned” activity was tightly correlated with excitatory receptor expression, with 73% of excitatory LPeD1 cells showing “patterned” activity based on the above criteria. In contrast, only 46% of biphasic LPeD1 and 10% of inhibitory LPeD1 fulfilled the criteria. These data demonstrate that “patterned” activity, as defined by our two above parameters may be required for the functional expression of excitatory nAChRs.

### Activity was necessary for the expression and maintenance of excitatory nAChR expression

**Figure 5:**
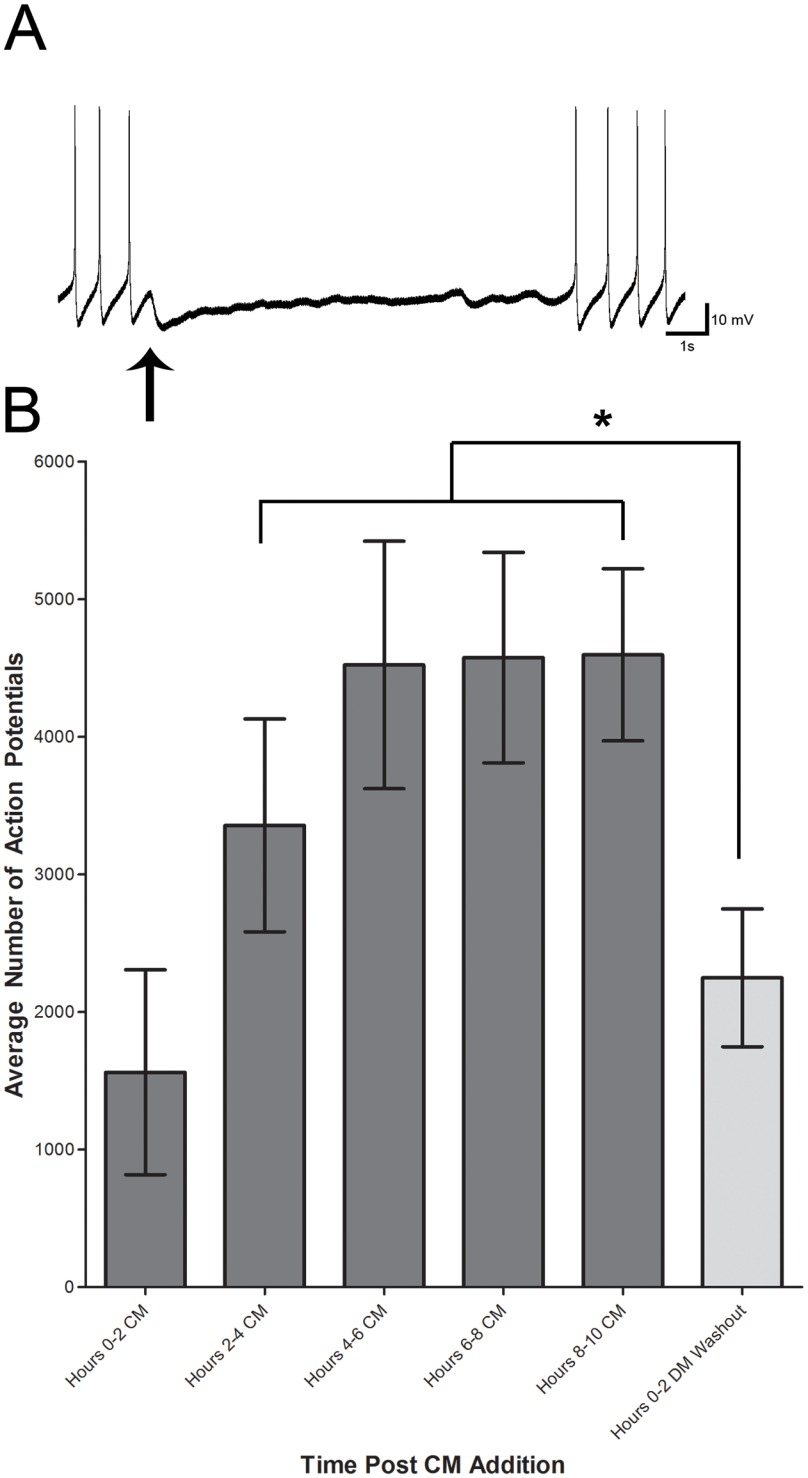
LPeD1 hyperpolarization inhibits excitatory nAChR expression and the presence of trophic factors is necessary to maintain activity in LPeD1. To determine whether activity is important for the expression of excitatory nAChRs, an intracellular sharp-electrode was used to inject hyperpolarizing current into LPeD1 neurons maintained in CM. (A) We found that the neurons hyperpolarized for 8 hours remained robust as demonstrated by their ability to elicit action potentials. However after blocking trophic factor induced activity, the LPeD1 neurons failed to express excitatory nAChRs despite being exposed to CM. Exogenous application of ACh (thin arrow) confirmed this by showing a reduction in spontaneous activity with each application. (B) Next, to determine whether the maintenance of activity in LPeD1 is CM dependent, the solution was washed out with DM after 10 hours of CM exposure. We found that following the CM withdrawal, LPeD1 activity significantly decreased to a level similar to that observed in the initial two hours following CM exposure. Significant differences are denoted with an asterisk (*) and is assumed if p < 0.05.

To determine whether activity triggered by CM was indeed necessary for the expression of excitatory nAChRs, we used intracellular sharp electrodes to selectively hyperpolarize LPeD1 neurons that were exposed to CM. Neurons were impaled with sharp electrodes and a hyperpolarizing current was injected to prevent action potentials from firing. Following the eight hours hyperpolarization period and neuronal quiescence, we first found that cells remained physiologically viable and as robust as their control counterparts (Fig. 5A). Using exogenous ACh application, we found that while 95% (n = 20) of control neurons showed an excitatory response to exogenous ACh puffs, only 29% (n = 7) of hyperpolarized neurons exhibited an excitatory response with the remaining 71% exhibiting an inhibitory response (Fig. 5A). These data thus demonstrates that the activity triggered by CM is necessary for the expression of functional excitatory nAChRs.

Next, to determine whether the triggered activity was important for the maintenance of functional excitatory AChR expression, we washed out the CM following 10 hours of exposure and observed changes in activity patterns. We found that CM withdrawal and its replacement with DM significantly decreased activity immediately after the removal of the trophic support. Specifically, activity recorded in the two hour window following the removal of CM was 2248 ± 501, which was significantly reduced (p < 0.05) compared to the time periods 2–4 hours (3355 ± 773), 4–6 hours (4521 ± 900), 6–8 hours (4575 ± 764) and 8–10 hours (4595 ± 625) (n = 10; Fig. 5B). When tested with exogenous ACh following DM washout, all cells exhibited an inhibitory response. These experiments demonstrate that the activity induced by CM is not only necessary for triggering the functional expression of nAChR, but also required for the maintenance of these excitatory nAChRs.

### Trophic factor effects on LPeD1 required the presence of the cell body

**Figure 6:**
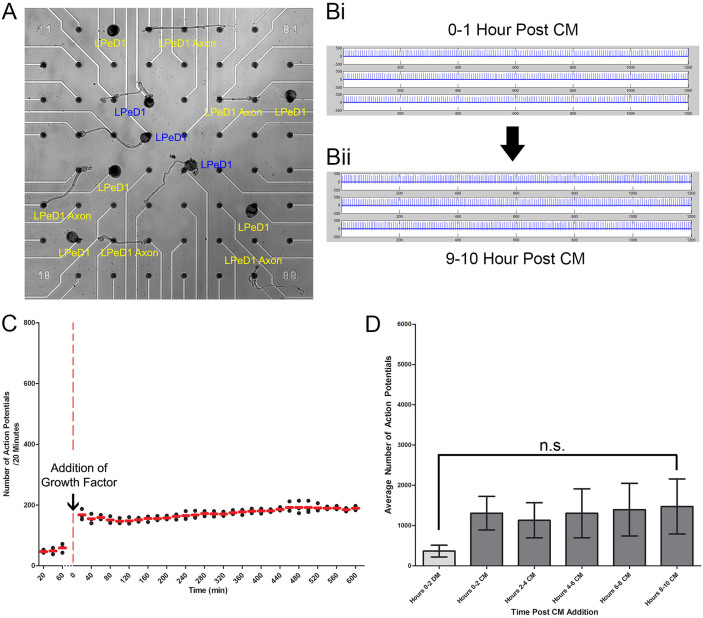
Trophic factor induced activity increase requires the somata. To test for trophic factor-induced activity in different cellular compartments (somal vs axonal) of LPeD1 neurons, we (A) cultured LPeD1 and its axons across multiple microelectrodes in DM, and severed the cell body from the axon. The resulting isolated axon was then monitored for electrical activity as CM was introduced (n = 4). We found that immediately following CM introduction, an (Bi) activity increase in the isolated axon was observed. However, unlike the activity pattern observed in the cell body, axonal activity (Bii) did not increase over time into the characteristic pattern seen in the cell body. Rather, the isolated axon maintained a (C) constant level of activity. (D) This increase was found to not be significantly different as determined by a one-way ANOVA, p > 0.05. Non-significantly different groups were designated with “n.s.”

Extrasomal compartments such as axons and dendrites are thought to exhibit trophic factor sensitivities, independent of their respective somata. Here, to determine the responsiveness of LPeD1 soma versus extrasomal (axonal) compartment to CM, we physically isolated the axon from the cell body, and tested its responsiveness to CM. To do this, neurons were extracted from the central ring ganglia with an extensive portion of their axons attached (~500 μm). The cell bodies were placed onto individual microelectrodes and their respective axons were gently laid across multiple adjacent electrodes. A fine sharp glass electrode was used to sever the cell body from its axon, resulting in an isolated somata and axon (Fig. 6A). We found that when in DM, compared to the LPeD1 soma (which had little to no activity), the isolated LPeD1 axons exhibited a low level of tonic activity (n = 4). When CM was subsequently introduced, the level of tonic activity in the isolated axons increased (Fig. 6Bi). However, unlike LPeD1 cell body, no change in patterned activity was observed in the axon (Fig. 6Bii). A scatterplot demonstrates the temporal pattern of initial activity increases, which rapidly reached a steady state level (Fig. 6C). When a one-way ANOVA was performed on the activity observed in the 2 hour bins, the increase was found not to be significantly different from that observed prior to CM exchange (Fig. 6D). These data thus suggest that the CM-induced, long-term activity-dependent changes specifically impact somata and may thus require *de novo* protein synthesis and gene induction for the functional expression of nAChR.

### *De novo* protein synthesis is required for activity changes following trophic factor addition

**Figure 7:**
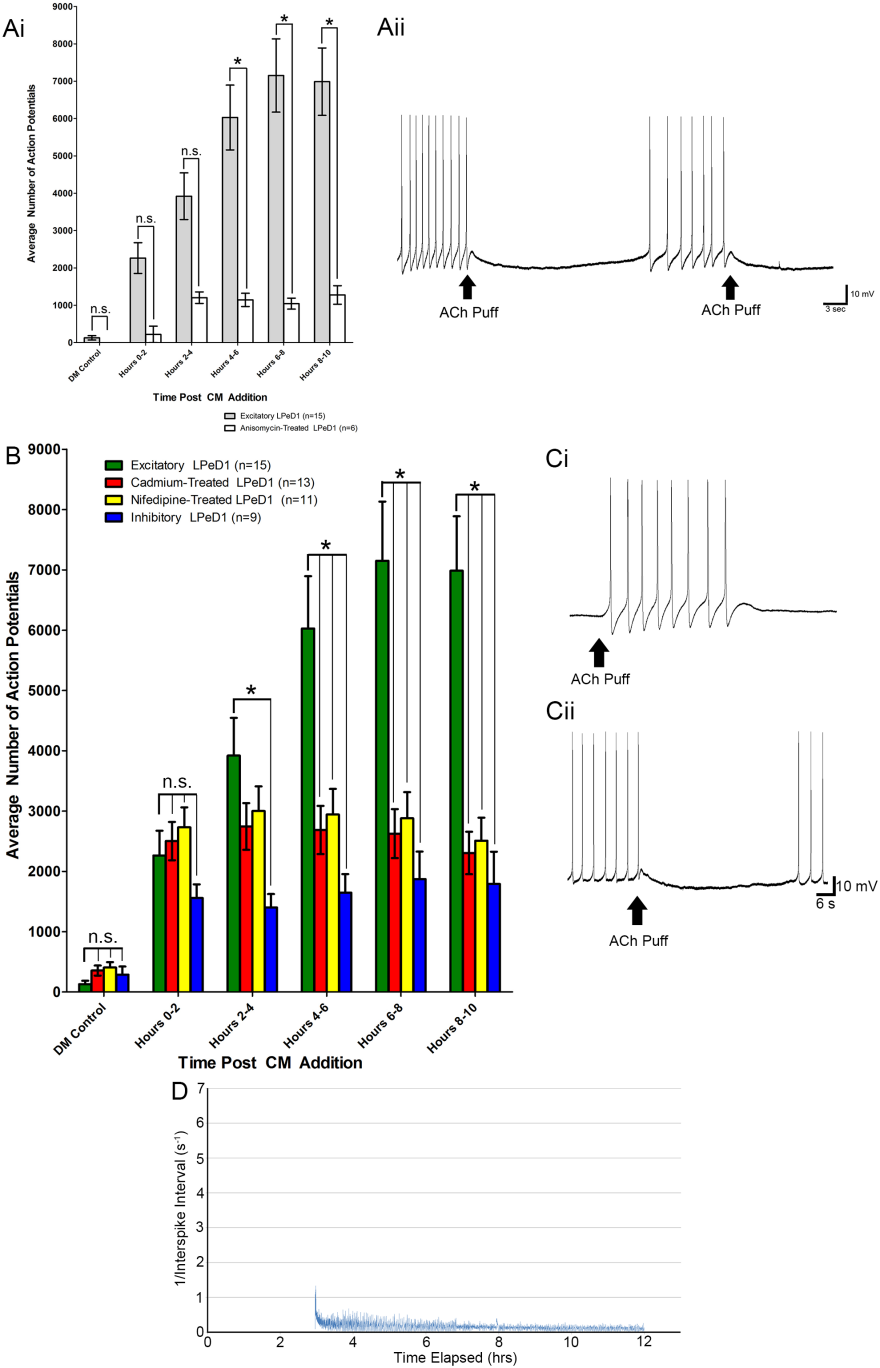
Perturbation of *de novo* protein synthesis and voltage gated calcium channel activity inhibits CM induced activity in LPeD1 neurons. The role of protein synthesis in long-term changes in activity/patterns induced by CM was investigated using the protein synthesis blocker anisomycin. (Ai) The activity of anisomycin treated LPeD1 cells was compared to controls over a period of 10 hours. In the presence of anisomycin, neurons failed to exhibit changes to their activity in response to CM. Specifically, activity was significantly reduced in hours 4–6, 6–8 and 8–10 in LPeD1 neurons exposed to anisomycin plus CM. (Aii) When exogenous acetylcholine was pressure applied onto these neurons, all anisomycin treated LPeD1 neurons expressed inhibitory AChRs. Next, (B) calcium channels on LPeD1 exposed to CM were blocked either with cadmium or nifedipine. While no significant difference in activity was seen in the 4 hours following CM addition, a significant reduction in activity levels was noted in the 5–10 hour range. This reduction in activity resembled that seen in inhibitory LPeD1 neurons. (Ci) Exogenous ACh application to control LPeD1 neurons showed an excitatory response, demonstrating the presence of excitatory nAChRs. However, in (Cii) nifedipine treated LPeD1 neurons, exogenously applied ACh exhibited an inhibitory response, suggesting the lack of an excitatory nAChR expression. (D) The graphical representation for 1/ISI showed that nifedipine prevented the development of patterned activity. Significance is denoted with an asterisk (*) and is assumed if p < 0.05. Non-significantly different groups were designated with “n.s.”

Next, we examined potential mechanisms underlying the CM-dependent modulation of activity. New protein translation has previously been shown to be a requirement for the expression of excitatory nAChRs^25^. However, whether long-term changes in activity patterns are also contingent upon *de novo* protein synthesis is yet to be studied. To determine this, we cultured LPeD1 neurons on MEAs in DM (1 hour) containing a protein synthesis blocker, anisomycin (15 μg/mL), prior to CM addition. We found that while treatment with anisomycin did not significantly affect the initial activity increases normally associated with CM at 0–2 and 2–4 hour time points, it did block the activity increases normally seen at hours 4–6, 6–8 and 8–10 in CM (Fig. 7Ai). This suggests that *de novo* protein synthesis is required for the expression of stereotypical activity pattern seen in control LPeD1 neurons exposed to CM, but not for the induction of an immediate activity response. In addition, exogenous ACh application revealed that all anisomycin treated neurons exhibited an inhibitory response (Fig. 7Aii).

### Calcium channel function is required for the upregulation and expression of patterned activity following trophic factor addition

To determine whether longer-term changes in CM-induced activity patterns involved Ca^2+^ influx via the activation of voltage gated Ca^2+^ channels (VGCC), the channel activity was perturbed by a non-specific calcium channel blocker, cadmium, and a specific L-type calcium channel blocker, nifedipine^27^. We observed that treatment with either cadmium or nifedipine significantly reduced the total level of activity. Specifically, in excitatory LPeD1 neurons, 26349 ± 13461 action potentials were elicited during the 10 hour period following CM exposure. In contrast, LPeD1 neurons treated with cadmium and nifedipine had on an average, 12864 ± 6385 action potentials and 14063 ± 6160 action potentials, respectively. When this activity was binned into two-hour blocks, no significant difference between excitatory LPeD1 neurons and cadmium or nifedipine treated neurons was observed at hours 0–2 and 2–4. However, by hours 4–6, 6–8 and 8–10, there was a significant reduction in the average number of action potentials (p < 0.05; Fig. 7B). When the 1/ISI was plotted, we found that blocking VGCCs prevented the stereotypical activity pattern observed in excitatory LPeD1 neurons (Fig. 7D). This suggests that the Ca^2+^ influx through VGCCs is a requirement for subsequent, long-term increases in activity and pattern formation at later time periods following the CM exposure. Additionally, similar to what was observed in our previous studies, inhibiting Ca^2+^ influx through VGCCs perturbed the functional expression of excitatory nAChRs in CM exposed LPeD1 neurons (Fig. 7Ci, Cii).

### LPeD1 contact with presynaptic partner VD4 reduces trophic factor induced activity

**Figure 8:**
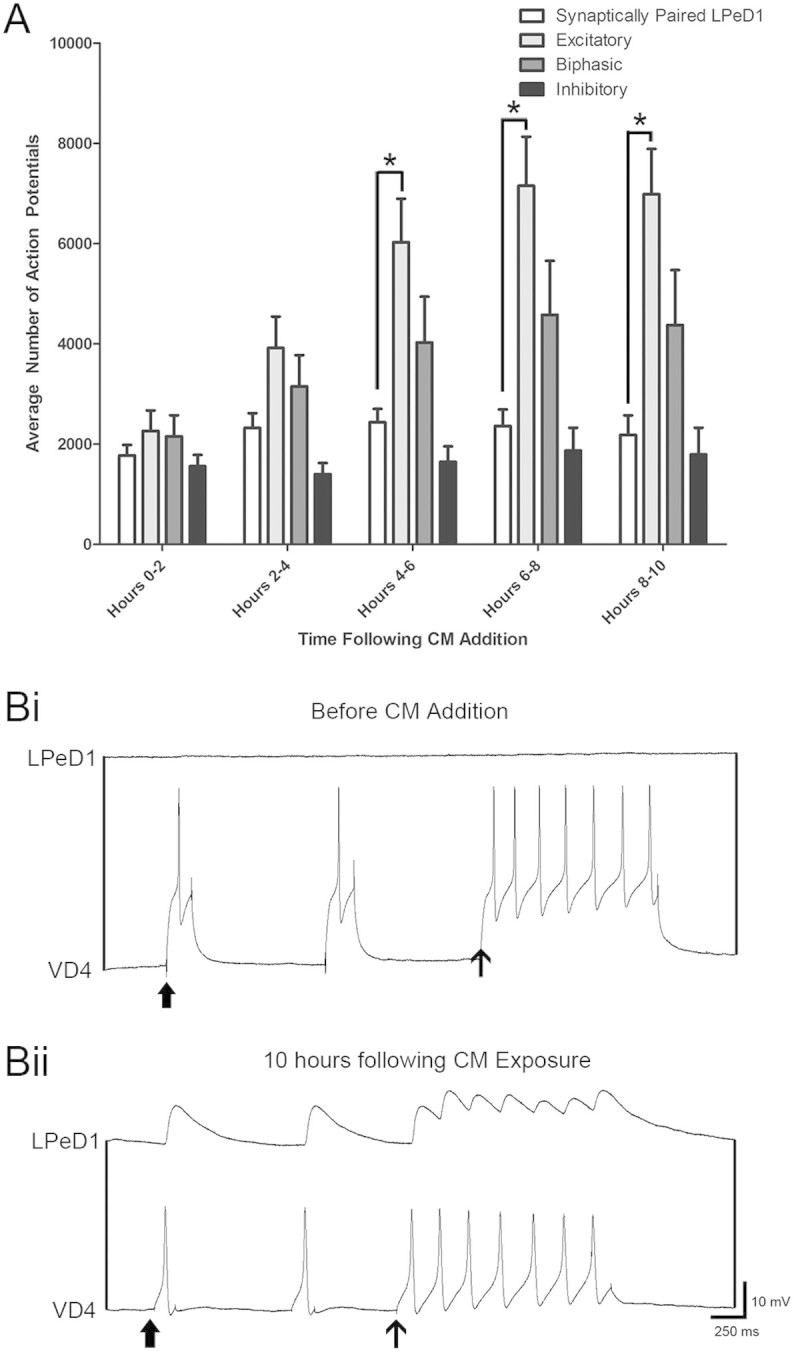
LPeD1 contact with presynaptic partner VD4 reduces trophic factor induced activity. To determine the effects of target cell contact on trophic factor induced activity, VD4 was paired overnight with LPeD1 in soma-soma configuration in DM (without trophic factors). (A) LPeD1 activity was then recorded in CM (with trophic factors) for a period of 10 hour. While the level of activity seen in the first 0–2 and 2–4 hours was not different from that seen in the single LPeD1 neurons exhibiting an excitatory response, it was, however, significantly reduced in hours 4–6, 6–8 and 8–10. The activity observed in hours 4–10 was not significantly different from that seen in inhibitory and biphasic single LPeD1 neurons, demonstrating that target cell contact reduced trophic factor induced activity in LPeD1. (Bi) Neurons were electrophysiologically tested for the presence of functional synapses. We found that neither single action potentials (thick arrow) nor a burst of tetanic spikes (thin arrow) elicited any response in postsynaptic LPeD1, suggesting that no functional synapse had formed in the DM. (Bii) Following CM exposure for 10 hours, a single action potential (thick arrow) elicited a 1:1 EPSP while a tetanic pulse (thin arrow) resulted in compound EPSPs, demonstrating the presence of a functional excitatory synapse. Significant differences are denoted with an asterisk (*) and significance is assumed if p < 0.05.

Next, to test whether pairing with a synaptic partner would alter trophic factor-induced activity patterns in LPeD1 neurons, presynaptic VD4 was juxtaposed with LPeD1 neurons in a soma-soma configuration in DM. The synaptic pairs were cultured on a high density microelectrode array (MCS-60HDMEA30/10iR-ITO-gr), permitting independent channel recordings of both pre- and postsynaptic neurons. Prior to CM addition, activity was recorded for a 30 minute time period to determine its basal levels. Similar to the activity seen in single isolated VD4 and LPeD1 neurons, a very low level of activity was observed in both synaptic partners. A 30 minute control recording of LPeD1 and VD4 neurons revealed an activity level of 1 ± 4 AP and 1 ± 3 AP (n = 12), respectively. Both neurons were then impaled with intracellular sharp-electrodes and evidence for synaptic connectivity sought electrophysiologically. Neither a single action potential nor the tetanic burst elicited in VD4 generated a corresponding postsynaptic response in LPeD1, indicating the absence of a functional synaptic connection between the cells (Fig. 8Bi). Next, DM was exchanged with CM and activity was further measured for 10 hours. Similar to the responses seen in single neurons, we found that CM triggered an initial increase in activity. In contrast, however, this activity pattern tended to follow a trend similar to that observed in inhibitory and biphasic neurons rather than excitatory LPeD1 cells. For instance, within the first two hours following trophic factor addition, the paired LPeD1 neurons exhibited 1772 ± 732 AP, a value statistically similar to single excitatory LPeD1 neurons (2263 ± 1594) (p < 0.05). At hours 4–6, where a significant increase in activity was observed in single excitatory LPeD1 neurons (6027 ± 3361), synaptically paired LPeD1 neurons continued to express a level of activity that was statistically similar to inhibitory and biphasic neurons with 2436 ± 921 AP (p > 0.05), but significantly reduced (p < 0.05) compared to single excitatory LPeD1 neurons. Activity at the remaining 6–8 hours and 8–10 hours revealed similar trends (Fig. 8A). Both single and paired VD4 neurons did not exhibit any significant response to trophic factors (data not shown). At the end of a 10 hours period, in all VD4/LPeD1 pairs, an excitatory synapse had formed whereby presynaptic activity triggered 1:1 postsynaptic potentials and a tetanic pulse elicited compound postsynaptic potentials (Fig. 8Bii).

Taken together, the above data demonstrates that: (1) VD4 and LPeD1 neurons when paired in DM behave similar to single, unpaired cells, (2) CM induced activity in post- but not the presynaptic neuron, and (3) target cell contact prevented the “runaway” activity in LPeD1 cells seen in single cells after CM exposure.

## Discussion

This study further underscores the importance of neurotrophic factors in eliciting unique patterns of activity, which are not only neuron, but also pairing status specific. We also showed that the trophic factor-induced activity patterns are prerequisite for the functional expression of nAChRs and synaptogenesis. We demonstrated, for the first time, that neurotrophic factors evoke a unique “signature” of electrical activity postsynaptically, which begins within minutes of neuronal exposure, lasts for hours and corresponds well with the functional expression of excitatory nAChRs. This study is also the first to relate various patterns of activity to the functional expression and maintenance of excitatory nAChRs.

The hypothesis that patterned, spontaneous activity is important for brain circuit formation stems from the Hebbian model which postulates that repeated stimulation of postsynaptic neurons by their presynaptic partners may result in the strengthening of the synapse^28^. Extensive work in retinal^29^ and cochlear projections^30^ have demonstrated the importance of spontaneous activity and also specific patterns of activity in facilitating circuit development. However, almost all studies to date have focused on presynaptic activity patterns and the activity patterns over an extended period of time during various developmental time frames have not been fully defined. In this study, we provide the first direct evidence that trophic factors acting exclusively on the postsynaptic cell trigger an activity pattern that primes the neuron for excitatory synapse formation—independent of presynaptic activity. It is also important to note that CM induced two unique activity patterns; one being an immediate triggering of action potential, followed by a transition to more sustained bursting over an extended time period. The immediate changes were observed in both the somata and its isolated axon, whereas the bursting pattern required somata, *de novo* protein synthesis and VGCC activity. These data thus demonstrate that although trophic factors may induce immediate global changes in activity, it is the pulsatile Ca^2+^ entry through voltage gated channels that may underlie gene induction required for the expression of excitatory nAChR. It therefore stands to reason that perhaps during cholinergic circuit development and plasticity, the brain may follow synaptogenic rules that are analogous to those of the neuromuscular junction, whereby the muscle defines synaptic sites independent of presynaptic neuron. Further development and refinement of these synaptic sites would, however, rely upon presynaptic signaling and the trophic factors present in the extracellular milieu^31^.

In this study, we showed that the cell body and *de novo* protein synthesis are crucial for an immediate enhancement of activity and its transition from low levels to a more persistent bursting pattern. While the precise nature of synthesized proteins remains elusive, there is strong indication that it may involve *de novo* synthesis of Ca^2+^ channels, which in turn may contribute towards sustained bursting pattern that we observed. Alternatively, Ca^2+^ entering through these channels, or its activated signaling cascade, may act as an inductive signal underlying the synthesis of new nAChR^32^. Accordingly, when we blocked Ca^2+^ channels with cadmium or nifedipine, both the bursting activity and the functional nAChR expression were perturbed.

The idea that the Ca^2+^ influx through VGCC into the cytoplasm activates gene expression is well established. Pulsatile Ca^2+^ entry has been established as an important trigger for controlling the levels of postsynaptic receptor expression and even regulating cell cycle events such as mitosis and apoptosis^33^. Any increase in activity may thus lead to an enhancement of intracellular calcium, which is then detected by calcium-binding proteins such as calmodulin or even directly by transcription factors to alter gene expression^32^,^34^. With a progression from low activity to persistent bursting seen in LPeD1 neurons, the amount of Ca^2+^ entering the cytoplasm may thus be sufficient to trigger downstream mechanisms mediating molecular transcription and translation. In support, we found that neurons with high variance activity did not express excitatory receptors, suggesting that the observed stereotypical activity pattern seen in excitatory LPeD1 neurons may be the optimal frequency required to maintain intracellular Ca^2+^ levels sufficient for downstream signaling cascades. The above notion supports the idea that a minimal Ca^2+^ “threshold” achieved by a unique pattern of activity “signature” is required for the expression (single) and consolidation (paired cells) of nAChRs.

A *Lymnaea* homologue of the multiple endocrine neoplasia type 1 (MEN1) tumor suppressor gene that encodes for the transcription factor menin was previously shown to be required in the postsynaptic neuron for proper synapse formation^35^. More recently, it was demonstrated that LPeD1 neurons treated with nifedipine and exhibiting inhibitory nAChRs could switchover to excitatory nAChRs—following the injection of synthetic *Lymnaea* MEN1^36^. These studies suggest that the molecules activated downstream of the Ca^2+^ signaling cascade may involve the activation of *Lymnaea* MEN1. Further studies are, however, needed to reveal a direct correlation between the activity patterns to specific MEN1 mRNA expression levels.

Interestingly, in single cells, trophic factors set the patterns of activity in a “runaway” mode while LPeD1 pairing with its synaptic partner resulted in “scaling down” of this activity to a level similar to that seen in DM. It appears that in single cells, a high level of patterned activity might serve to sustain the expression of excitatory nAChR, priming the neurons for immediate synaptogenesis upon contact with its partner cells. In contrast, in paired cells, synaptic partnership and receptor localization at the contact sites may eliminate the need for a “runaway” activity-mediated expression of nAChRs, thus scaling down both the activity and the receptor expression. This notion is consistent with observations made at the neuromuscular junction (NMJ), whereby contacts between motor neurons and their synaptic muscle partners result in synaptic site specific localization of nAChRs and a down regulation of extra-synaptic receptor expression^37^,^38^. In summary, our data provides further unique insights into trophic factor mediated actions on neuronal activity and its specific regulation of nAChR expression. Based on the data presented here, we propose that trophic factors are essential not only for the developmental expression but also the maintenance of nAChRs during synaptic plasticity underlying learning and memory. In the absence of trophic factors and the ensuing perturbed activity, the cholinergic networks will be rendered dysfunctional as we see during neurodegenerative diseases such as Alzheimer disease^39^,^40^,^41^. Whereas several studies have demonstrated a predominant presynaptic locus of neurotrophin action on cholinergic neurons, a similar postsynaptic role is also plausible.

## Methods

### Animals

The fresh water snail, *Lymnaea stagnalis*, was maintained at room temperature (20–21°C) in an aerated aquarium with filtered water. The animals were fed a consistent diet of romaine lettuce. Younger animals approximately 1–2 months old (18–20 mm) were used for neuronal cultures and older snails approximately 2–6 months old (25–30 mm) were used in the preparation of CM.

### Cell Culture

A detailed cell culture protocol is published elsewhere (Syed, et al.)^42^. Briefly, central ring ganglia from 1 to 2 month old animals were removed and treated with trypsin (2 mg/mL; T-4665; Sigma-Aldrich, St Louis, MO, USA) for 20 min followed by trypsin inhibitor (2 mg/mL; T-9003; Sigma-Aldrich) for 15 min. Identified neurons LPeD1 and VD4 were isolated by gentle suction applied through a fire-polished and Sigmacote® treated glass pipette (SL2; Sigma-Aldrich). The cells were then plated on individual electrodes of a poly-L-lysine coated microelectrode arrays (MEA; Multichannel Systems; Reutlingen, Germany) in defined media (DM; L-15 Special Order; Life Technologies, Gaithersburg, MD, USA). The neurons were allowed to settle overnight and used for experiments the next day (12–18 hours post culture). Trophic factors containing *Lymnaea* conditioned medium (CM) were obtained by incubating isolated *Lymnaea* central ring ganglia from 2 to 6 month old animals with a shell length of 25–30 mm for 3–6 days in defined medium (DM – no added trophic factors).

### Neuronal Activity Recordings and Analysis

The electrical activity of neurons cultured on the MEAs (Multichannel Systems) was recorded through an MEA amplifier and PCI acquisition card (MEA1060; Multichannel Systems). Activity recordings were made from the neurons for 2 hours in DM, with a break in recording to perform acetylcholine receptor assay (see below) followed by a switch to CM and 10 further hours of recording before another acetylcholine receptor assay was performed (see below). During the 2 hour DM recordings, a DM to DM switch was performed to control for any possible mechanically induced activity. The recordings were processed using MC_Rack software (Multichannel Systems) and individual action potentials with their associated timestamps were extracted. The output from MC_Rack was then processed by Excel (Microsoft; Redmond, WA, USA) to yield the number of action potentials per hour as well as interspike intervals (ISI; time elapsed between two adjacent action potentials). The inverse of the ISI was taken to generate frequency plots and to provide longitudinal visualization of activity pattern. The plots were then scored in a blinded fashion to determine whether or not the activity was patterned and a subsequent prediction of their excitatory status was performed.

### Acetylcholine Receptor Assay

Functional expression of acetylcholine receptors was tested via pulsed application of acetylcholine (1 μM; A-112; Research Biochemicals; Natick, NA, USA) at 12 psi for 500 ms through glass microelectrodes with an opening diameter of 5 μm mounted on a pressure injection system (PV800; World Precision Instruments; Sarasota, FL, USA). Concomitant intracellular recording was made to detect change in membrane potential and to determine functional, excitatory nAChR response. As mentioned above, the receptor expression of the cells was tested before and after trophic factor exposure. Intracellular recordings were conducted using glass microelectrodes (1.5 mm internal diameter; World Precision Instruments) with resistances between 20 and 50 MΩ. The microelectrodes were filled with a saturated solution of K_2_SO_4_ and used to impale the neurons using micromanipulators (M0-103; Narashige, Tokyo, Japan) on an inverted microscope (Axiovert 100 TV; Zeiss, Thornwood, NY, USA) or an upright microscope (Olympus BX61WI; Olympus, Richmond Hill, ON, Canada). The electrical signals were amplified with an intracellular recording amplifier (IR-283; Cygnus Technology, Delaware Water Gap, PA, USA), sent through a digitizer (Digidata 1322A; MDS Inc, Toronto, Canada), and recorded on Axoscope 10.2 (MDS Inc).

### Pharmacological Agents

All chemicals were obtained from Sigma-Aldrich unless stated otherwise. All chemicals were dissolved in DM at a concentration such that the added volume into CM was less than 1% of total CM volume. In the experiments with the protein synthesis blocker anisomycin (12.5 ug/mL; A9789; Sigma-Aldrich) the cells were pre-incubated with anisomycin in DM prior to CM addition. Cadmium was used at a final concentration of 100 μM and nifedipine at 10 μM.

### Statistical Analysis

Pearson's chi-squared test was used to test for significant correlations between receptor expression phenotype and patterned activity as well as anisomycin treatment and patterned activity. Univariate analysis of variance (ANOVA) was used to analyze all other studies. Tamhane's correction was used to correct for unequal variance in the anisomycin studies. All statistical tests were performed with SPSS 19.0 for Windows (IBM; Armonk, NY, USA) and significance was assumed if p values were less than 0.05 (p < 0.05).

## Author Contributions

C.L., A.J.L. and N.S. wrote the main manuscript text. C.L. and A.J.L. prepared figures 1–4. C.L., P.W. and J.A. prepared figures 5–8. Experiments were designed by C.L., A.J.L., P.W. and N.S. Data was analyzed by A.L. and N.W. Experiments were carried out by C.L., A.J.L., P.W. and W.Z. All authors reviewed the manuscript.
